# The SARS-CoV-2 Reproduction Number R_0_ in Cats

**DOI:** 10.3390/v13122480

**Published:** 2021-12-10

**Authors:** Jose L. Gonzales, Mart C. M. de Jong, Nora M. Gerhards, Wim H. M. Van der Poel

**Affiliations:** 1 Department of Epidemiology, Bioinformatics & Animal Models, Wageningen Bioveterinary Research, 8200 AB Lelystad, The Netherlands; nora.gerhards@wur.nl; 2 Quantitative Veterinary Epidemiology, Wageningen University, 6708 PB Wageningen, The Netherlands; mart.dejong@wur.nl (M.C.M.d.J.); wim.vanderpoel@wur.nl (W.H.M.V.d.P.); 3 Department Virology & Molecular Biology, Wageningen Bioveterinary Research, 8200 AB Lelystad, The Netherlands

**Keywords:** SARS-CoV-2, cats, transmission, reproduction number

## Abstract

Domestic cats are susceptible to SARS-CoV-2 virus infection and given that they are in close contact with people, assessing the potential risk cats represent for the transmission and maintenance of SARS-CoV-2 is important. Assessing this risk implies quantifying transmission from humans-to-cats, from cats-to-cats and from cats-to-humans. Here we quantified the risk of cat-to-cat transmission by reviewing published literature describing transmission either experimentally or under natural conditions in infected households. Data from these studies were collated to quantify the SARS-CoV-2 reproduction number *R_0_* among cats. The estimated R_0_ was significantly higher than one, hence cats could play a role in the transmission and maintenance of SARS-CoV-2. Questions that remain to be addressed are the risk of transmission from humans-to-cats and cats-to-humans. Further data on household transmission and data on virus levels in both the environment around infected cats and their exhaled air could be a step towards assessing these risks

## 1. Introduction

A relevant concern in the control of the ongoing Covid-19 pandemic is the risk domestic animals could play in the maintenance and transmission of SARS-CoV-2. Assessing this risk implies quantifying transmission from humans-to-animals, from animals-to-animals and from animals-to-humans. Large epidemics in farmed minks have confirmed this risk for that specific species [1]. The role of cats is of particular interest because they are in close contact with humans and frequently in contact with other cats. Available field [2,3,4,5,6,7,8,9,10] and experimental data [11,12,13,14,15] indicate that cats are susceptible to infection, occasionally show mild clinical signs and may be able to transmit the infection between cats. Indeed, transmission experiments confirmed this possibility [11,12,13,14,15], however, the lack of a proper statistical assessment of transmission in the reported experiments limits the confident extrapolation of the results from the experiment to the population.

An important question when assessing the risk of transmission is whether cat-to-cat transmission can be sustained. A key measure to answer this question is the basic reproduction number *R_0_*, which is the average number of individuals to whom a typical infectious individual will transmit the infection in a naive population. *R_0_* is a key parameter in infectious disease epidemiology, it provides an indication of the transmissibility of a pathogen and the risk of epidemic transmission. When *R_0_* > 1, one can expect sustained transmission with risk of a major outbreak and endemicity to occur, whereas when *R_0_* < 1 the infection is likely to peter out [16]. Other parameters which contribute to quantitatively describe transmission are: (1) the latent period *L*, which is the time from becoming infected to becoming contagious, (2) the infectious period *T*, which is the average period of time an individual is contagious and (3) the transmission rate parameter *β* which is the number of contact infections caused by one typical infectious individual per unit of time.

Here, published experiments and observational studies describing infection and transmission of SARS-CoV-2 between cats were reviewed. Data from these studies were collated and analysed to statistically confirm whether cat-to-cat transmission can be sustained and to provide estimates of relevant transmission parameters.

## 2. Materials and Methods

### 2.1. Literature Search and Selection of Studies

#### 2.1.1. Search Question

For our research question, we defined the study population as naive domestic cats exposed, experimentally or naturally within a household, to SARS-CoV-2. The intervention of interest was infection via experimental challenge or household infection and the outcomes of interest were cat-to-cat transmission and longitudinal monitoring of shedding in infected cats.

#### 2.1.2. Search Strategy

We searched the electronic databases PubMed, EMBASE, BioRxiv, and MedRxiv as indexed by the ‘COVID Open Access Project’ [17]. We filtered the dataset of COVID-19 related research based on keywords in the title and or abstract: “((cats) OR (feline) AND (transmission) OR (transmissibility) OR (passage) OR (susceptibility))”. The search was performed on 9 August 2021. Additionally, we checked the reference lists of the included studies for relevant publications.

#### 2.1.3. Study Selection and Data Extraction

The first screening and selection was made based on titles and abstracts. Only experimental studies and observational studies in line with the research question were included. The second step consisted of full-text screening of the selected studies. For experimental studies, studies that included naive sentinels exposed directly or indirectly to infected (inoculated) cats to assess transmission were selected for data extraction. For observational studies, selected studies for data extraction were reports in which detailed information of infection of household cats were provided at the household level. Studies with one cat or multiple cats per household were selected for data extraction.

Data was extracted either from the text, figures or the supplementary data files. For studies that did not provide the full data, we used WebPlotDigitizer [18], to extract the data from the figures. Data on the first and last time of positive RNA or virus detection during the infection process, peak virus concentration (units depended on the laboratory method used), the number of cats per experimental group or household and the number of infected cats at the end of the study were extracted for analysis. A detailed description of data extraction and preparation is provided in Section 2.2.

### 2.2. Data Analysis Methods for the Estimation of Transmission Parameters

#### 2.2.1. Data Preparation

##### Transmission Experiments

The daily presence of infection in individual cats as measured by virus shedding in nasal (swabs or washes) [11,12,13,14,15] or faecal samples [14], from inoculated and contact infected cats was extracted from the published articles. Within each experimental group (inoculated + contact) a cat was classed as infectious when it was shown as shedding virus, regardless of the viral load. Contact cats were considered susceptible for the period of days before the first day (after estimation of the latent period) they were shown to shed the virus. For data collation, a latent period (time from infection to becoming infectious) of one day was used. One day was used because the latent period was estimated to be 1.1 (95% Confidence intervals (CI): 0.5–2.2) days for contact-infected cats and 0.84 (95% CI: 0.5–1.4) days for inoculated cats, with no significant difference observed in the latent period between contact-infected and inoculated cats (Table 3). In Table S1 it is shown how these data were prepared.

For estimation of the mean peak of shedding, the highest virus load recorded for each animal during the experiment period was extracted for analysis. Data preparation for the estimation of the latent and infectious period is explained below and shown in Table S2.

##### Household Observations (Observational Studies)

For the assessment of transmission, data from households housing more than one cat were selected. From the selected households, the following data were extracted: number of people living in the household (at least one person infected), the total number of cats in the household, the number of cats diagnosed as positive by RT-PCR and serology as well as the last day that the cat was tested for serology (Table S3).

For the estimation of the peak and length of shedding (Infectious period), data from households housing one or more infected cats which were longitudinally followed after first diagnosis were extracted for analysis. Table S4 shows how these data were extracted and prepared for analysis.

#### 2.2.2. Estimation of the Transmission Rate β (Day^−1^)

The transmission rate *β* could only be estimated using the data from the transmission experiments. For this analysis detailed information on the infection and transmission process in time is required. This level of detail was not available for the households.

For analysis, each experimental group (mostly pairs, all small-scale experiments) of cats was considered as an independent trial, and data was collated in the form of the number of Infectious (I), Susceptible (S), and new Cases (C) within a Time interval (d*t*) of one day (Table S1). These data were analysed using a generalised linear model (GLM) with a binomial error distribution and a cloglog link. Given that *β* is the transmission rate parameter per unit of time *t*, then the probability of new infection (cases) *p* is

(1) $$p=1-exp\left(-\beta \frac{I}{N}t\right)$$

which upon linearisation gives

(2) $$log\left(-log\left(1-p\right)\right)=log\left(\beta \;\right)+log\left(\frac{I}{N}t\right)$$

To fit the GLM, $log\left(\frac{I}{N}t\right)$ was introduced as an offset variable. The exponent of this model intercept, log(*β*), is the estimated transmission rate parameter *β (day^−1^)*.

#### 2.2.3. Estimation of the Latent and Infectious Period

The length of the latent L and infectious period T was quantified by performing a parametric survival analysis where different distributions were assessed. The distributions that best fitted the data (judged by the model with lowest AIC) were an exponential distribution for L and a Weibull distribution for T. Because data from the households were left censored, resulting in an uncertain time (mostly >5 days) of initial exposure of the first infected cat to their infected owners (Table S4), the latent period using household data could not be estimated. The estimation of the infectious period was possible, however, the left censoring for most observations was not considered in the survival model, because the models would not converge. Hence, the estimates are likely an underestimation of the infectious period, when using RT-PCR positive as a correlate of infectiousness.

Tables S3 and S4 show how the data were prepared for this analysis and in Table S5 the estimated parameters of the Weibull distributions of T are provided.

#### 2.2.4. Estimation of the Reproduction Number R_0_

The reproduction number *R_0_* was estimated as the product of *β* and *T* (Only for transmission experiments). The *95*% confidence intervals for *R_0_* were derived by Monte Carlo (MC) simulations (1000 replications) assigning to *β* and *T* lognormal and Weibull distributions, respectively. Parameters for these distributions were obtained from the same data set when estimating these parameters as explained above. *R_0_* was also estimated by the final size method (FSM) [19]. This method, different from estimating *R_0_* as a result of *β*×*T*, does not require detailed temporal information of the infection and transmission process. The FSM only uses the information of the number of infected individuals at the end of the epidemic, when there are either no more infectious or susceptible individuals in the population. The FSM used for analysis is described in detail elsewhere [20,21]. To simplify the analysis of transmission using the household data, it was assumed that the source of infection of secondarily infected cats was the first infected cat (infected by the owner) in the household and the contribution of infected owners to the infection of secondarily infected cats was not included in the analysis.

Assessment of *R_0_* > 1 was performed using the FS method and by MC sampling (when estimating *R_0_ = β* × *T*).

#### 2.2.5. Statistical Software

All analyses were performed using the statistical software *R* [22]. The library Survival was used for the survival analysis. A detailed description of how to prepare data from a transmission experiment and the R codes for analysis has been reported elsewhere [23].

## 3. Results

### 3.1. Literature Search and Selected Studies

The literature search, after deduplication, provided 154 studies (149 identified for the searched databases and five by checking reference lists). Thirty-nine [2,3,4,5,6,7,8,9,10,11,12,13,14,15,24,25,26,27,28,29,30,31,32,33,34,35,36,37,38,39,40,41,42,43,44,45,46,47,48], studies were then selected for full-text screening. Finally, 16 studies, five experimental studies [11,12,13,14,15] and 11 observational studies [4,5,6,7,8,9,25,26,43,47,48], were selected for data extraction and analysis.

In Table 1 and Table 2, the experimental and household studies included for analyses are summarised. Of the experimental studies, four [11,12,13,15], assessed direct-contact transmission and one [14] indirect (droplet/aerosol) transmission. These studies used different study designs with respect to age and the number of inoculated (donor) and contact cats included within an experimental group. All experiments used inoculation doses ≥10^5^ PFU (Gaudreault et al. [13] used 10^6^ TCID_50_) and the predominant inoculation route was intra-nasal inoculation. Following inoculation, infection and transmission were monitored by longitudinally detecting and measuring virus shedding in nasal, faecal or oropharyngeal samples collected from inoculated and contact- or droplet-infected cats. The laboratory methods used to monitor infection were either virus isolation (VI) [11,12] or RT-PCR [13,14]. From the observational studies, data from 18 households housing infected people and at least one infected cat were included for analysis. Twelve of these households [4,5,6,7,8,9,43,47], had either two, three or four cats, one household was a shelter housing 22 cats [43] and five households [25,26] had only one cat (Table 2 and Tables S3 and S4). The infection process of owners and cats was longitudinally followed in most of these households.

### 3.2. Cat-to-Cat Transmission Parameters

For all experiments, *L* was estimated to be about one day, with no significant differences observed between inoculated and contact infected cats (Table 3). Mean peak virus shedding estimated for the different experiments ranged from 10^3.5^ to 10^4.1^ PFU/mL of processed sample or 10^3.4^ to 10^9.0^ RNA copies/mL. Samples from households were only processed by RT-PCR with reported shedding levels as high as 10^8.5^ RNA copies/swab sample or RT-PCR CT values as low as 21 (Table S4). The estimated mean peak shedding was 10^6.1^ RNA copies/mL (Table 3).

The type of test has a clear influence on the estimation of *T*, with estimates performed using RT-PCR data leading to an overestimation of *T* and consequently *R_0_* when compared with the FSM estimates (Table 3, see estimates for Bao et al. [15]). Using VI data from contact-infected cats (assumed to closely reflect a “natural” infection) to estimate *T* and the corresponding *R_0_* led to similar estimates to those found using the FSM (Table 3). The experimental design had a large influence on the estimation of *β;* with the design used in two of the studies [12,13], leading to an overestimation of this parameter and large standard errors. Although a small sample size was used, the pair-transmission design used by Shi et al. [14], Halfmann et al. [11] and Bao et al. [15] allowed the estimation of *β* and *R_0_* with good certainty*_._* The former experiment assessed droplet-transmission whilst the latter two experiments assessed direct transmission and allowed confirmation that *R_0_* is significantly higher than 1 (*p* < 0.05). When combining these two experiments, the estimated *R_0_* (*T* * *β*) for cats was 3.0 (95%CI: 1.5.–5.8) or 3.3 (FSM) (95%CI: 1.1–11.8). Using data from households, the estimated *R_0_* (FSM) was 2.3 (95%CI: 1.1–4.9) (Table 3). Based on the overlapping 95%CI, it can be concluded that the household and experiments estimates of *R_0_* do not differ significantly. Similarly, the estimates of *T* and virus shedding levels from household data were similar to those estimates from the experiments (Table 3).

Experimental data were also used to quantify droplet/aerosol transmission. Compared to direct transmission, droplet/aerosol transmission was slower *β =* 0.14 (95%CI: 0.02–0.44) *day*^−1^ and may happen to a lower extend *R_0_* = 1.0 (95%CI: 0.2–4.7) than direct transmission (Table 3).

## 4. Discussion

By using both data from experimental studies assessing cat-to-cat transmission [11,12,13,14,15] and data from studies that followed cats from infected households [4,5,6,7,8,9,43,47,48], we statistically confirmed that sustained transmission of SARS-CoV-2 among cats can be expected (*R_0_* > 1). To put this into perspective, scenarios in which contacts between stray and household cats take place [3], could lead to the persistence of the virus in the cat population. As the pandemic is still ongoing in humans and cats do not get clinically ill, transmission in cats can easily go unnoticed. Epidemic transmission between cats as seen for example in farmed minks [1], may not occur everywhere but it may occur in some shelters or clusters of feral cat [3,43] populations. Therefore, it is important that surveillance efforts are addressed to monitor not only infection but also transmission in the cat population. Surveillance studies where infection is assessed and reported at the household level, such as those included for analysis in this study, would contribute to having a better assessment of the transmission risk from owners to their pets, between pets, and if possible, from pets to their owners.

This study shows the importance of quantitatively assessing transmission when performing transmission experiments and the relevance of a proper experimental design to obtain reliable estimates of different parameters that describe the transmission process. By combining field and experimental observations we could partly validate the suitability of a pair-transmission design to study transmission and the validity of the estimated parameters. The estimated virus shedding levels, duration of shedding (*T*) and *R_0_* using experimental data were similar to estimates performed using household observations. Noting the assumptions made for the analysis of household data (see below), the results indicate that pair-transmission experiments appear to provide a close approximation of the expected transmission dynamics of SARS-CoV-2 between cats at the household level.

Whilst field observations would provide the ideal data to assess transmission, it is practically impossible to obtain detailed temporal data to have a thorough understanding of the transmission dynamics. Given this limitation, in order to analyse the household data, we had to make assumptions that influence our estimates. The main assumption being that secondarily infected cats were infected by the first infected cat in the household, ignoring the possibility of these cats becoming infected by contact with the infected owner. As a result, the *R_0_* estimates could be overestimated. As for *T* and shedding levels, observations were left censored, since the first diagnosis of the cats was around five to seven days after the clinical onset of the infected owner (Table S4) and not all cats were followed daily, which may affect the accuracy of these estimates. Nevertheless, they were similar to the experimental estimates. The combination of experimental and field data in this study improved the characterisation of transmission between cats and increased the certainty in the estimated parameters.

An important parameter to describe transmission is *T*. The estimation of this parameter is dependent on the type of sample (respiratory or anal sample or both) used as a correlate of infectiousness. Whilst we do not exactly know which are the main transmission routes between cats, we could speculate that the cat’s behaviour and way of housing influence transmission. In addition to close exposure to each other’s respiratory secretions, “butt sniffing” is a natural and important form of cat-to-cat communication, and cats within a household may usually share a litter box. Given these behavioural and housing traits, both respiratory [14] and anal shedding may contribute, in different degrees, to cat-to-cat transmission of SARS-CoV-2.

Considering both that infected cats shed high levels of virus, and that droplet/aerosol transmission is possible, the risk for cat-to-human transmission of SARS-CoV-2 may not be low. There is a need to further investigate this risk. Experimental assessment of, for example, the probability of transmission via a contaminated environment around an infected cat and measurements of virus concentrations in infected cats’ exhaled air would provide further information to quantify the risk for cat-to-human transmission. This data combined with more detailed transmission and environmental contamination data [5,26] from infected household cats could aid in further quantifying the combined risks of human-to-cat and cat-to-human transmission. A thorough understanding of the transmission of SARS-CoV-2 at the human–animal interplay is important to obtain a better insight into the population dynamics of this virus.

## Figures and Tables

**Table 1:** Summary of the experimental procedures showing the study design, the age of the cats, the inoculation route and dose, the type of samples taken and the diagnostic method used to quantify virus levels in time.

Study	Type of Transmission	Design I ×S ^1^	Cat’s Age (Months)	Inoculation Route	Dose (log_10_)	Units ^2^	Sample (Route) ^3^	Diagnostic Test
Halfmann et al. [11]	Direct contact	1 × 1	3.5–4.2	Nasal, Tracheal, Oral, Ocular	5.7	PFU	Respiratory	VI ^4^
Bosco-Lauth et al. [12]	Direct contact	2 × 2	60–96	Nasal	5.4	PFU	Respiratory/rectal	VI
Gaudreault et al. [13]	Direct contact	3 × 1	4.5–5	Nasal, Oral	6	TCID_50_	Respiratory	RT-PCR
Bao et al. [15]	Direct contact	1 × 1	8–18	Nasal	6	TCID_50_	Respiratory/rectal	RT-PCR
Shi et al. juveniles [14]	Indirect-droplet/aerosol	1 × 1	2.3–3.3	Nasal	5	PFU	Respiratory	RT-PCR
Shi et al. subadults [14]	Indirect-droplet/aerosol	1 × 1	6–9	Nasal	5	PFU	Rectal	RT-PCR

^1^ I = number of inoculated cats and S = number of susceptible contacts per group at the start of the experiment. ^2^ PFU = Plaque-forming units, TCID50 = Fifty-percent tissue culture infective dose. ^3^ Type of samples considered as respiratory were: nasal swabs, oropharyngeal swabs, nasal washes. Rectal samples were: rectal swabs or faeces. ^4^ VI = Virus Isolation.

**Table 2:** Summary description of the households studies included for estimation of the shedding (infectious) period and the Reproductive Number R0.

Studies ^1^	No. of Households	Total No. of Cats per Household	Number of Households with > 1 Cat Infected ^2^	Sample (Route) ^3^	Diagnostic Test	Data Used for the Estimation of
Chaintoutis et al. [4], Hamer et al. [9], Neira et al. [6]	3	3	1	Respiratory/Rectal	PCR, Serology	*R*_0_, Shedding
Hamer et al. [9], Klaus et al. [5], Segales et al. [8], Neira et al. [6] Goryoka et al. [7], Jara et al. [43], Keller et al. [47]	8	2	6	Respiratory/Rectal	PCR, Serology	*R*_0_, Shedding
Barrs et al. [25], Bessiere et al. [26], Garigliany et al. [48]	5	1		Respiratory/Rectal	PCR, Serology	Shedding
Jara et al. [43]	2	4, 22 ^4^	1 ^4^	Blood	Serology	*R*_0_

^1^ Complete extracted data from each of these studies is provided as supplementary Tables S3 and S4. ^2^ For a cat to be considered infected it had to be seropositive the last time the cats in the household were sampled. ^3^ Type of samples considered as respiratory were: nasal swabs, oropharyngeal swabs or oral swabs. Rectal samples were: rectal swabs or faeces. When serological tests were performed blood samples were taken. ^4^ This was a shelter with 22 cats, out of which 8 were seropositive. No transmission was observed in the household with 4 cats.

**Table 3:** Quantified parameters for direct contact and droplet transmission of SARS-CoV-2 between cats using data from transmission experiments or observational studies describing infection and transmission at household level ^1^.

Study	No. Groups/Households (No. without Transmission)	Peak Shedding (log_10_ x/mL) ^2^ Mean ± SD	Latent Period *L* (Days) ^3^Mean (95% CI)	Infectious Period *T* (Days) ^3^ Mean (95% CI)	Transmission Rate *Β* (Day^−1^) Mean (95% CI)	*R_0_* Mean (95% CI)	
						*T* × *β*	Final Size
*Direct transmission*							
Halfmann et al. [11]	3 (0)	4.0 ± 0.5 PFU ^4^ 3.5 ± 0.6 PFU ^5^		4.6 (3.0–5.7) ^4^ 5.4 (3.6–6.8) ^5^	0.64 (0.11–1.66)	2.9 (1.0–7.6)	>1.2
Bosco-Lauth et al. [12]	1 (0)	4.0 ± 0.6 PFU ^4^ 4.1 ±1.4 PFU ^5^		6.8 (4.5–8.4) ^4^ 4.7 (3.0–5.8) ^5^	2.77 (0.45–8.93)	15.2 (4.4–50.9)	
Gaudreault et al. [13]	2 (0)	9.0 RNA ^4^		6.6 (3.8–8.7) ^4^	1.46 (0.23–5.04)	9.6 (2.7–33.1)	
Bao et al. [15]	8 (4)	3.4 ± 0.5 RNA ^4^ 4.9 ± 0.6 RNA ^5^		10.0 (6.5–12.4) ^4^ 11.6 (7.5–14.4) ^5^	0.69 (0.21–1.65)	6.8 (2.8–11.3)	2.0 (0.5–7.7)
Combined			1.1 (0.5–2.2) ^4^ 0.8 (0.3–1.9) ^5^	4.6 (3.0–5.7) ^4^	0.67 (0.28–1.31) ^7^	3.0 (1.5–5.8)^6^	3.3 (1.1–11.8) ^7^
*Droplet*/*aerosol transmission*							
Shi et al.juveniles [14]	3 (2)	7.0 ± 0.3 RNA ^5^		8.1 (4.6–10.6) ^5^	0.10 (0.01–0.46)	0.8 (0.2–4.4)	1.0 (0.1–7.6)
Shi et al. subadults [14]	3 (2)	4.9 ± 0.4 RNA ^5^		5.7 (3.3–7.5) ^5^	0.22 (0.01–0.99)	1.2 (0.2–6.7)	1.0 (0.1–7.6)
Combined			0.8 (0.3–1.9)^5^		0.14 (0.02–0.44)	1.1 (0.3–3.6)^8^	1.0 (0.2–4.7)
*Household transmission*							
Households [4,5,6,7,8,9,43,47,48]	13 (3)	6.8 ±1.2 RNA ^9^ 5.1 ± 1.7 RNA^9^		7.4 (2.3–14.2) ^9^ 5.6 (1.8–10.8) ^9^			2.3 (1.1–4.9)

^1^ Where relevant, empty cells represent analysis not performed. Data was not suitable/sufficient to perform the corresponding analysis. ^2^ x values are plaque-forming units (PFU), RNA copy numbers. CT = Real-time PCR (RT-PCR) cycle threshold. SD = standard deviation. ^3^ *L* was estimated fitting an exponential distribution. *T* was estimated fitting a Weibull distribution using either virus isolation data or PCR data (see column peak shedding) (Text S2). CI = Confidence Intervals. ^4^ These are estimates for the contact-infected cats. ^5^ These are estimates for the inoculated-infected cats. ^6^ Estimates performed combining data from the different studies or groups when a combined analysis was possible. For estimation of *R*_0_ the estimated *T* from the contact infected cats from Halfmann et al. [11] was used. This was because contact-infected cats were assumed to resemble “natural” infection better than inoculated cats and that virus isolation is a better indicator of infectiousness than RT-PCR. ^7^ These estimates were performed combining the data from Halfmann et al. [11] and Bao et al. [15]. Data from these experiments were combined for these analyses because their similar experimental design (pair-transmission experiments). ^8^ Estimated using the estimated *T* from the juvenile group. This estimate was based on nasal shedding. ^9^ The upper estimates are for respiratory/oral samples and the lower ones for rectal/fecal samples. Estimated Peak shedding reported in Ct values were 27.7 ± 5.5 for Respiratory/oral samples and 32.1 ± 1.2 for rectal/fecal samples. No differences in peak shedding or *T* were observed between respiratory/oral and fecal/rectal shedding.

## Data Availability

The data used for analysis and presented in this study are available within the manuscript and in the Supplementary Tables S1–S5.

## Supplementary Materials

The following are available online at https://www.mdpi.com/article/10.3390/v13122480/s1, Table S1: Collated data for the quantification of the transmission rate β (day^−1^). Data for each pair of cats (inoculated + contact) was collated daily from day one post inoculation to the day the contact cat was assumed infected (one day before shedding virus). Table S2: Collated data for the estimation of the infectious and latent periods. Table S3: Collated data from infected households with more than one cat. These data were used for the estimation of the reproductive number R_0_ using the final size method. Table S4: Collated data from observational studies describing the longitudinal follow up of infection in infected cats from infected households. These data were used to estimate the duration of observed shedding in naturally infected cats. Table S5: Estimated Weibull parameters (Shape and Scale) describing the length of the infectious period T.
