# Supplementary material for: The SARS-CoV-2 Reproduction Number R0 in Cats
Source: Viruses. 2021 Dec 10;13(12):2480. doi: 10.3390/v13122480 (PMC8704225; doi:10.3390/v13122480)
Supplement: Supplementary file 1 [file viruses-13-02480-s001.zip › Table S1.pdf]

Table S1. Collated data for the quantification of the transmission rate β (day^{-1}). Data for each pair of cats (inoculated + contact) was collated daily from day one post inoculation to the day the contact cat was assumed infected (one day before shedding virus).[a]

Reference	Group	Day	S	I	C	N	dt
Halfmann et al.[11]	1	1	1	1	0	2	1
Halfmann et al.[11]	1	2	1	1	1	2	1
Halfmann et al.[11]	2	1	1	1	0	2	1
Halfmann et al.[11]	2	2	1	1	0	2	1
Halfmann et al.[11]	2	3	1	1	0	2	1
Halfmann et al.[11]	2	4	1	1	0	2	1
Halfmann et al.[11]	2	5	1	1	1	2	1
Halfmann et al.[11]	3	1	1	1	0	2	1
Halfmann et al.[11]	3	2	1	1	0	2	1
Halfmann et al.[11]	3	3	1	1	0	2	1
Halfmann et al.[11]	3	4	1	1	1	2	1
Gaudreault et al.[13][b]	1	1	1	3	0	4	1
Gaudreault et al.[13]	1	2	1	3	1	4	1
Gaudreault et al.[13]	2	1	1	3	1	4	1
Bosco-Lauth et al.[15][c]	1	0	2	2	0	4	0.5
Bosco-Lauth et al.[15]	1	1	2	2	2	4	0.5
Bao et al.[15]	P01	1	1	1	1	2	2
Bao et al.[15]	P02	1	1	1	1	2	2
Bao et al.[15]	P03	1	1	1	1	2	2
Bao et al.[15]	P04	1	1	1	1	2	2
Bao et al.[15]	P11	1	1	1	0	2	2
Bao et al.[15]	P12	1	1	1	0	2	2
Bao et al.[15]	P13	1	1	1	0	2	2
Bao et al.[15]	P14	1	1	1	0	2	2
Shi et al. Subadults.[14]	1	1	1	1	1	2	2
Shi et al. Subadults.[14]	2	1	1	1	0	2	2
Shi et al. Subadults.[14]	2	2	1	1	0	2	2
Shi et al. Subadults.[14]	3	1	1	1	0	2	2
Shi et al. Subadults.[14]	3	2	1	1	0	2	2
Shi et al. Juveniles.[14]	1	1	1	1	0	2	1
Shi et al. Juveniles.[14]	1	1	1	1	0	2	2
Shi et al. Juveniles.[14]	1	3	1	1	1	2	2
Shi et al. Juveniles.[14]	2	1	1	1	0	2	1
Shi et al. Juveniles.[14]	2	1	1	1	0	2	2
Shi et al. Juveniles.[14]	2	3	1	1	0	2	2
Shi et al. Juveniles.[14]	2	5	1	1	0	2	2
Shi et al. Juveniles.[14]	2	7	1	1	0	2	1
Shi et al. Juveniles.[14]	3	1	1	1	0	2	2
Shi et al. Juveniles.[14]	3	3	1	1	0	2	2
Shi et al. Juveniles.[14]	3	5	1	1	0	2	2
Shi et al. Juveniles.[14]	3	7	1	1	0	2	1

[a] Group = identifies the experiment group, Day = day post inoculation, S = number of Susceptible at the start of the day, I = number of infectious, C = number of new infections “cases” confirmed the next day, N = total number of animals in the experiment, dt = time interval in days.

[b] Samples were taken every two days. And all contacts were already positive two days post challenge. When using a dt = 2 (only one row per group), it was not possible to estimate β. Hence to be able to analyse this data, we had to assume that one contact in for instance group 1 became infected at day 2 and the other contact in group 2 at day 1. In this way we used a dt = 1.

[c] Both contacts were infected the next day post challenge. By using a dt = 1 (only one row), it was not possible to estimate β. Hence, to be able to make estimations, we divided the day in two periods (dt = 0.5) and assumed transmission took place during the second period.
