# Supplementary material for: The SARS-CoV-2 Reproduction Number R0 in Cats
Source: Viruses. 2021 Dec 10;13(12):2480. doi: 10.3390/v13122480 (PMC8704225; doi:10.3390/v13122480)
Supplement: Supplementary file 1 [file viruses-13-02480-s001.zip › Table S2.pdf]

Table S2. Collated data for the estimation of the infectious and latent periods.[a]

Reference	Treatment	Shedding	Time	Time2	Event	LPtime	LPtime2	Event_Lp	Peak shedding	Units
Halfmann et al.[11]	Inoculated	nasal	6	6	1	1	1	1	3.6	PFU
Halfmann et al. [11]	Inoculated	nasal	4	4	1	2	2	2	2.8	PFU
Halfmann et al. [11]	Inoculated	nasal	6	6	1	1	1	1	4	PFU
Halfmann et al. [11]	Contact	nasal	5	5	1	1	3	3	4.5	PFU
Halfmann et al. [11]	Contact	nasal	4	4	1	5	5	2	4.1	PFU
Halfmann et al. [11]	Contact	nasal	5	5	1	4	4	2	3.5	PFU
Bosco-Lauth et al.[12]	Inoculated	nasal	4	4	1	1	1	1	3.9	PFU
Bosco-Lauth et al.[12]	Inoculated	nasal	4	4	1	1	1	1	4.3	PFU
Bosco-Lauth et al.[12]	Inoculated	nasal	4	4	1	1	1	1	3.7	PFU
Bosco-Lauth et al.[12]	Inoculated	nasal	5	5	0	1	1	1	6.3	PFU
Bosco-Lauth et al.[12]	Inoculated	nasal	5	5	0	1	1	1	2.3	PFU
Bosco-Lauth et al.[12]	Contact	nasal	6	8	3	1	1	1	3.6	PFU
Bosco-Lauth et al.[12]	Contact	nasal	6	8	3	1	1	1	4.4	PFU
Gaudreault et al.[13]	Contact	nasal	7	11	3	1	3	3	9	RNA
Gaudreault et al.[13]	Contact	nasal	4	4	1	1	3	3	9	RNA
Shi et al. Subadults.[14]	Inoculated	faeces	5	7	3	3	3	2	5.3	RNA
Shi et al. Subadults. [14]	Inoculated	faeces	5	7	3	3	3	2	4.9	RNA
Shi et al. Subadults. [14]	Inoculated	faeces	5	7	3	3	3	2	4.5	RNA
Shi et al. Juveniles. [14]	Inoculated	nasal	6	8	3	2	2	2	7.5	RNA
Shi et al. Juveniles. [14]	Inoculated	nasal	8	10	3	2	2	2	7.5	RNA
Shi et al. Juveniles. [14]	Inoculated	nasal	8	10	3	2	2	2	7.9	RNA
Bao et al. [15]	Inoculated	nasal	11	11	1	1	1	1	4.5	RNA
Bao et al. [15]	Inoculated	nasal	11	13	3	1	1	1	5.6	RNA
Bao et al. [15]	Inoculated	nasal	11	13	3	1	1	1	4.3	RNA
Bao et al. [15]	Inoculated	nasal	13	13	1	1	1	1	5.2	RNA
Bao et al. [15]	Contact	nasal	9	11	3	2	2	2	4	RNA
Bao et al. [15]	Contact	nasal	11	13	3	2	2	2	3.7	RNA
Bao et al. [15]	Contact	nasal	5	7	3	2	2	2	2.8	RNA

Bao et al. [15]	Contact	nasal	9	11	3	2	2	2	3.1	RNA

[a] Each row represents observations for an individual cat. Time = number of days the cat was assumed infectious (shedding virus) and detected positive for the last time; Time2 = time in days when cat was first negative; event = categorical variable where 1 = cat recovered (shedding stopped), 0 = cat was still shedding at the end of the experiment (right censoring) or 3 = shedding stopped between time and time2. LPtime, LPtime2 and LPevent are the data used for the estimation of the latent period. LPtime = day animal last detected negative (starts shedding), LPtime2 = animal start shedding, LPevent = categorical variable where 1 = event observed at time 1, 2 = left censoring, 3 = shedding started between LPtime and LPtime2.
