# Supplementary material for: The SARS-CoV-2 Reproduction Number R0 in Cats
Source: Viruses. 2021 Dec 10;13(12):2480. doi: 10.3390/v13122480 (PMC8704225; doi:10.3390/v13122480)
Supplement: Supplementary file 1 [file viruses-13-02480-s001.zip › Table S3.pdf]

Table S3. Collated data from infected households with more than one cat. These data were used for the estimation of the reproductive number R_0 using the final size method.

Study	Household id	No. of humans	No. of cats	PCR+ cats	Serology + cats[a]	Last day serum sample[b]
Chaintoutis et al.[4]	1	1	3	2	1	63
Hamer et al.[9]	D	NP[c]	2	2	2	93
Hamer et al. [9]	OO	NP	3	3	3	38
Klaus et al.[5]	2	2	2	1	1	36
Segales et al.[8]	3	1	2	2	2	10
Neira et al.[6]	1	NP	2	1	1	39
Neira et al.[6]	2	2	3	3	1	40
Goryoka et al.[7]	2	3	2	2	2	20
Jara et al.[43]	A	NP	2		2	NA
Jara et al. [43]	C	1	22		8	84
Jara et al. [43]	D	NP	2		2	21
Jara et al. [43]	F	NP	4		1	70
Keller et al.[47]	1	1	2		2	35

[a] Only cats serology positive (+) were considered infected for estimation of R_0.

[b] This is the time from the day of the first cat or human confirmation of infection in the household and the time the last serum sample for serology was taken from all cats in the household. These data help to confirm the assumption that the transmission process within each household reached its end (no more infectious or susceptible cats present in the household).

[c] NP = not provided
