# Supplementary material for: The SARS-CoV-2 Reproduction Number R0 in Cats
Source: Viruses. 2021 Dec 10;13(12):2480. doi: 10.3390/v13122480 (PMC8704225; doi:10.3390/v13122480)
Supplement: Supplementary file 1 [file viruses-13-02480-s001.zip › Table S4.pdf]

Table S4. Collated data from observational studies describing the longitudinal follow up of infection in infected cats from infected households. These data were used to estimate the duration of observed shedding in naturally infected cats.[a]

Study	Houshold ID	Cat ID	Sample	Cat's Age (Years)	Time	Time2	Event	Owner symptoms (Days)[a]	Maximum observed shedding	Units
Chaintoutis et al.[4]	1	C2	Oropharyngeal	10	7	7	1	-7	7	log_{10}RNAcopies/swab
Chaintoutis et al.[4]	1	C3	Oropharyngeal	10	7	7	1	-7	8.5	log_{10}RNAcopies/swab
Chaintoutis et al.[4]	1	C2	Fecal	10	5	5	1	-7	5.6	log_{10}RNAcopies/swab
Chaintoutis et al.[4]	1	C3	Fecal	10	7	7	1	-7	6.6	log_{10}RNAcopies/swab
Barrs et al.[25]	1	DHS/7/F	Nasal	7	11	11	1	-10, -1	6.3 (21.3)[d]	log_{10}RNAcopies/swab
Barrs et al.[25]	1	DHS/7/F	Oral	7	7	9	3	-10, -1	5.6 (22.9)	log_{10}RNAcopies/swab
Barrs et al.[25]	1	DHS/7/F	Rectal	7	1	1	1	-10, -1	3.2 (33)	log_{10}RNAcopies/swab
Barrs et al.[25]	2	AHS/13/M	Oral	13	4	9	3	NP[c]	22	Ct[c]
Barrs et al.[25]	3	SSH/5/M	Oral	5	5	10	3	NP	26.8	Ct
Neira et al.[6]	1	Cat 1	Nasal	10	5	5	1	-7, -6	31	Ct
Neira et al.[6]	1	Cat 1	Fecal	10	4	4	1	-7, -6	30.8	Ct
Neira et al.[6]	1	Cat 2	Nasal	10	17	17	1	-7, -6	21.9	Ct
Neira et al.[6]	1	Cat 2	Fecal	10	9	9	1	-7, -6	31.2	Ct
Neira et al.[6]	1	Cat 3	Nasal	10	7	10	3	-7, -6	29.9	Ct
Neira et al.[6]	1	Cat 3	Nasal	10	7	7	1	-7, -6	29.9	Ct
Bessiere et al.[26]	3	1	Oropharyngeal	3	2	2	1	-5	35.6	Ct
Garigliany et al.[48]	1	1	Oropharyngeal	15	7	7	1	-10	35.7	Ct
Garigliany et al.[48]	1	1	Fecal	15	9	9	1	-10	33.2	Ct

[a] For the estimation of the duration of shedding, each row represents observations for an individual cat within a household. The following columns were used: Time = number of days the cat detected RT-PCR positive and detected positive for the last time; Time2 = time in days when cat was first negative; Event = categorical variable where 1 = cat recovered [shedding stopped, no censored data], 0 = cat was still shedding at the end of the experiment [right censoring] or 3 = shedding stopped between Time and Time2.

[b] This is number of days between the onset of clinical signs in the cat's owner[s] [multiple values, represent multiple people in the household] and the first detection of infection in the cat. A value of -7 means the owner first had clinical signs 7 days before positive confirmation of infection in the cat. For estimation of the duration of shedding this left censoring was ignored and duration of shedding was considered from the first day of detection of infection until the last day that the cat was detected positive by RT-PCR. Hence Estimates of duration of shedding [as measured by PCR] are likely to be underestimated.

[c] Ct = RT-PCR cycle threshold. NP = data not provided.

[d] Data in brackets are the RT-PCR Ct values.
