# Supplementary material for: The SARS-CoV-2 Reproduction Number R0 in Cats
Source: Viruses. 2021 Dec 10;13(12):2480. doi: 10.3390/v13122480 (PMC8704225; doi:10.3390/v13122480)
Supplement: Supplementary file 1 [file viruses-13-02480-s001.zip › Table S5.pdf]

Table S5. Estimated Weibull parameters (Shape and Scale) describing the length of the infectious period *T*.

Study	Infection route	Shape	Scale
Direct transmission			
Halfmann et al.[11]	Contact	7.8	4.8
	Inoculation	7.8	5.7
Bosco-Lauth et al.[12]	Contact	7.8	7.1
	Inoculated	7.8	4.9
Gaudreault et al.[13]	Contact	7.8	7.0
Bao et al.[15]	Contact	7.8	10.5
	Inoculated	7.8	12.2
Droplet transmission			
Shi et al. juveniles.[14]	Inoculated	6.6	8.2
Shi et al. subadults.[14]	Inoculated	6.6	5.8
Observational studies			
Household cats [4-9,43,47,48]	Contact	2.2	8.7[a]
		2.2	6.6[b]

[a] This estimate is made using data from respiratory/oral samples

[b] This estimate is made using data from rectal/fecal samples.
